# Virtual Goods Recommendations in Virtual Worlds

**DOI:** 10.1155/2015/523174

**Published:** 2015-03-05

**Authors:** Kuan-Yu Chen, Hsiu-Yu Liao, Jyun-Hung Chen, Duen-Ren Liu

**Affiliations:** Institute of Information Management, National Chiao Tung University, Hsinchu 30010, Taiwan

## Abstract

Virtual worlds (VWs) are computer-simulated environments which allow users to create their own virtual character as an avatar. With the rapidly growing user volume in VWs, platform providers launch virtual goods in haste and stampede users to increase sales revenue. However, the rapidity of development incurs virtual unrelated items which will be difficult to remarket. It not only wastes virtual global companies' intelligence resources, but also makes it difficult for users to find suitable virtual goods fit for their virtual home in daily virtual life. In the VWs, users decorate their houses, visit others' homes, create families, host parties, and so forth. Users establish their social life circles through these activities. This research proposes a novel virtual goods recommendation method based on these social interactions. The contact strength and contact influence result from interactions with social neighbors and influence users' buying intention. Our research highlights the importance of social interactions in virtual goods recommendation. The experiment's data were retrieved from an online VW platform, and the results show that the proposed method, considering social interactions and social life circle, has better performance than existing recommendation methods.

## 1. Introduction

Virtual worlds (VWs) are computer-simulated environments which allow users to create their own virtual characters as avatars. Through this avatar, they are able to interact with other users or objects [1]. In the VW, users not only communicate with others via email or chat, as on traditional SN websites, but also interact with other users via various activities in this visual environment. Every user has his/her virtual life in the VW, which includes buying virtual commodities to decorate his/her virtual home, making friends, visiting others' homes, creating families, joining clubs, and so forth. These activities form the user's social life circle. The avatar in VWs is a new virtual identity for users free from their identity in the real world or traditional social networks. Users can obtain more self-satisfaction, self-improvement, and release of pressure by building virtual lives, engaging in social life circles and playing games in VWs. The participation and behavior in these two worlds (real and virtual) share similarities yet also exhibit differences.

Since VWs provide not only game-based features but also a virtual life to live, users may decorate their own virtual space and buy products to enrich their lives in VWs. For users, the self-satisfaction gained from those activities impels them to spend more time on them; for the VW companies, increased users' cohesion and affection enhance users' purchase orientation and related discussions, thereby inducing more players to enter the field of the VW. Players' participation and behaviors are crucial in sustaining the VW.

In the VW, users can easily visit others' virtual houses, make friends, chat or leave messages, and cowork on activities with one click of a mouse. The visualized virtual goods in these interactions spur the related users' shopping intentions. This unique interaction characteristic of the VW helps users not only to interact with friends, but also to gain close connections with others. We call those who are not friends of target users but have contact with them, social circle neighbors.

With the rapidly growing user volume in VWs, platform providers launch virtual goods in haste and stampede users to increase sales revenue. However, the rapid developed virtual items become difficult to remarket. It not only wastes virtual companies' intelligence resources, but also makes it difficult for users to find suitable virtual goods to enrich their virtual home in daily virtual life. There have been fruitful research results in traditional goods recommendation methods, such as content-based filtering (CBF) [2], collaborative filtering (CF) [3–5], and hybrid filtering [6, 7]. However, there is no research which considers the special characteristics of virtual life, social interaction, and the buying behaviors derived from virtual worlds. Most of the existing studies on VWs focus on user behaviors. Guo and Barnes [8] and Domina et al. [9] discussed buying behaviors in VWs and analyzed the factors which influenced the behavior. However, there is no research which discusses virtual goods recommendations in VWs considering social influence.

This research proposes a novel virtual goods recommendation method, composed of two parts: the characteristics in virtual lives and the contact influence resulting from the social interactions in the VWs. In the former part the goal is to find users' virtual life features and social influence in regard to buying behavior. The virtual items in users' virtual homes demonstrate their current preferences and can be used to identify virtual life features. Those virtual life features can be used to find the users' features similar neighbors with high virtual life similarity (UVLS). In the latter part the aim is to calculate the contact influence of the high interactive users on the target users. The contact influence is based on contact strength and buying influence. The more interactions they have, the more contact strength between the two users exists. The buying influence is the degree of the social neighbors' effect in their buying decisions. The buying influence considers not only the perspective of the influenced user, but also the influential side to demonstrate the importance of such interactions.

In this research, we collected the dataset from a famous virtual world website (http://www.roomi.com.tw/) in Taiwan to evaluate the proposed model. This paper makes the following three contributions.

1. Our research highlights the importance of user interactions in the virtual goods recommendation. Frequent interaction with the non-friends is the special characteristic of VWs. The existing recommendation methods in the real worlds, based on similar users' preference or user's friends' comments, are not effective for goods recommendation in VWs.
2. This paper combines methods of determining goods similarity in existing studies and the contact influences we propose to recommend virtual goods. Virtual goods recommendations in VWs enable users to enhance the contact strength and buying influence by the shopping interactions with other users. As the users immerse in the virtual home they create, it helps to develop the stability among users and they become the loyal customers for VW providers.
3. The experiment data were retrieved from an online virtual world platform in Taiwan; the results show that the proposed method achieves better recommendation performance than existing methods do. The model is also deployed in this VW platform. The better the recommendation performance is, the better the user experience is and the more willing users are to stay in this VW; while users spend more time having fun, they tend to spend more money as well. This model helps both users and platform providers obtain a win-win solution.

In the following sections, we review the related works on VW and recommendation systems, as well as trust-based and social influence recommendation systems. Then, we elaborate on the method we propose and the experiment results that validate the proposed approach. The final section closes with some conclusions and suggestions for future work.

## 2. Related Work

The VW is a three-dimension space where everyone can play as an avatar to enjoy a variety of contacts and play games with others. There are studies discussing the phenomena and future implications in VWs, but few explore the recommendation systems. Existing recommendation systems bear fruitful results in regard to goods recommendation, and some even explore the dynamics behind players buying virtual goods, but few consider the users' interaction in virtual worlds.

In this section, we first refer to existing studies in the field of VWs and then elaborate on the recent research on recommendation systems to demonstrate the lack of virtual goods recommendation methodology in VWs and finally describe existing trust-based and social influence in recommendation systems.

### 2.1. Virtual Worlds

Castronova et al. define a VW as a persistent, synthetic, online environment that can be accessed by many users at the same time [10]. Messinger et al. [11] elaborate the background from electric games and social networks to VWs; they provide the history of VWs and future possibilities and discuss the difference between the real world and VWs. Utz [12] concluded that friends can share similar interests in VWs and that the main goals for VWs' users are to play online games and role-play. Besides, the users who spend more time in VWs often make more friends and also get more self-satisfaction.

### 2.2. Recommendation Systems

Recommendation systems involve a filtering technology based on the customers' preferences or interests which filters off the information the customer do not need and predicts product they may like. There are several domains which apply recommender systems, such as movies [4], documents and books [13–15], webpages [16], music [5], and other products [14, 17]. In general, there are some commonly used recommendation methods, including collaborative filtering (CF), content-based filtering (CBF), and hybrid filtering. In this study, we applied CF in part of the proposed model.

Collaborative filtering (CF) is a widely used recommendation method. It focuses on finding the relationship between items or users. There are two types of CF method: one is user-based CF and the other is item-based CF. The user-based CF focuses on identifying a group of users with similar interests for determining target users and makes recommendations for the target users based on the profiles of this group of users [4, 18, 19]. The item-based CF first considers the relationships between items and then computes the similarity among items to make recommendations for users based on similarity of these items [20]. Amazon [21] and MovieLens both apply the item-based CF method.

### 2.3. Trust-Based and Social Influence Recommender Systems

With the widely developed social networks, recommendation systems based on friendship or trust have become a significant topic in researches. With these trust relations, one can predict the trust degree between nonrelated users [22–24]. The trust degree is derived according to the interactions among users (e.g., rating) [25] and the friendship or the trust value that users describe directly. The friend list in Facebook indicates the trust relations of friends. The trust list in Epinions can be used to increase the accuracy of recommendations [26, 27].

The social influence concerns the users' influences in social networks and the user behaviors that are influenced by other users (e.g., friends) [28]. From blogs [29], tweets [30], and tags [31], we can find the influential users by mining the users' behaviors to estimate the influential level of recommenders. Weng et al. [32] estimate the influence of users on their followers by the relative amount of content that the followers received. Cui et al. [33] take the number of friends who click the target user's post to measure the target user's influence. Thus, some researches combine the social influence and the traditional recommendation method to recommend items [34]. The users' influence can be estimated by taking the ratio of the number of items that a target user received to the number of items a recommender recommended.

## 3. Virtual Goods Recommendation Approach

The VWs are similar to the real world and users can engage in heterogeneous activities in VWs. But the users' behavior patterns in VWs also differ from those of the real world. With the use of an avatar, users have more willingness to contact strangers and express their real selves. They need not worry about their real identities, so users are more apt to interact with strangers in VWs, such as talking to strangers, visiting strangers' virtual homes, and even sending gifts. Users are more easily influenced by others under these circumstances. Furthermore, users have their own virtual lives. They can establish a virtual life by doing various activities as in the real life. In this virtual life, users show their preferences through buying behaviors.

The social life circle neighbors are a group of people that users have visited, chatted with, or done activities with. They may not be the users' friends, but they are close to the users because of intensive interactions. The social life circle neighbors may influence users' preferences through different interactions, such as discussing fancy virtual goods, sending different kinds of furniture as gifts to users, or even just letting users visit their virtual homes. When users see the visual set of virtual goods, they may like some virtual goods they had not noticed before and change their preference in the future.

Different interactions may result in different levels of influence. Thus, the first step is to analyze the influence of different interactions. Then we can analyze the contact strength score with the social life circle neighbors. A higher score means that this neighbor is closer to the target user and may influence the target user's preferences and buying behaviors. Through this analysis, we can find some social life circle neighbors close to the target user who have more influence than other users. Also, we can design an analysis of virtual good buying behaviors based on their interactions.

In the VW, users buy virtual goods to build their virtual life, and users have their own unique setting of visualized goods. This unique setting of visualized goods is the user's virtual life feature. In this part, we analyze the virtual goods that the target user owns to find his/her preferences. Then we can detect other users, such as target user's neighbors, with similar virtual lifestyles. Similar users may buy virtual goods which are highly related.

**Figure 1:**
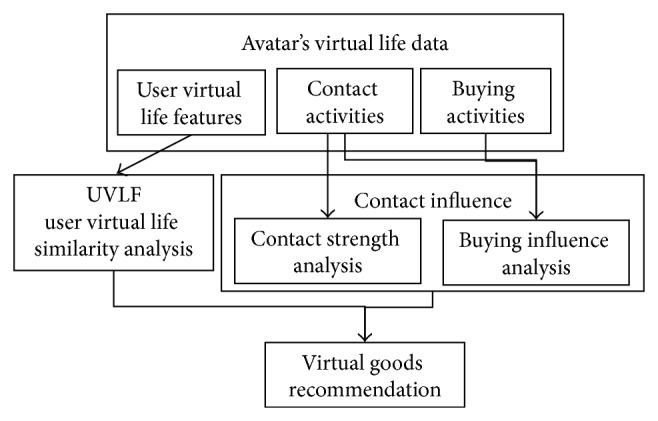
Overview of proposed approach.

In this work, we propose a virtual goods recommendation method based on users' virtual life features (UVLF) and contact influence in VWs.  Figure 1 illustrates the framework, which consists of several phases. First, we analyzed the similarity of users' virtual life features. Second, we focused on the interactions between target user *u* and his/her social circle life neighbor users. Users may have more influence on each other when they have more interactions. Thus, we analyzed the contact strength to discover closer users of the target user *u* and used this contact strength and the buying behaviors influence to weigh the contact influence. Finally, we integrated the similarity score and target user *u*'s contact influence from social life circle neighbors to recommend virtual goods.

### 3.1. User Virtual Life Similarity Analysis

The users in VWs build their virtual lives to reflect their distinguishing characteristics. Users buy and decorate their virtual homes through a set of items; we call them user virtual life features (UVLFs). UVLFs are unique and can be detected; they can be applied to analyze the similarity of users. The target user's preference was compared among contacted users to find his/her virtual life similar neighbors (*VlNbr*). The *VlNbr*'s preference will be part of recommendation composition.

Users seldom put different styles of items together because the virtual interior would look tacky. Due to the limited number of decorations, users may choose what they like most or what is most suitable for consistency. Therefore, the visual setting of items a user builds is the user's real-time preference. It is more suitable to analyze users' current preference by their visual setting of virtual goods than by their historic buying log. We use Jaccard Coefficient to compute the virtual life similarity (UVLS) of users *u* and *v*. For example, if user *u* has five items *i*_1_, *i*_2_, *i*_3_, *i*_4_, and *i*_5_; user *v* has three items *i*_1_, *i*_5_, and *i*_6_, then the UVLS between* users u* and *v* is 2/6. *UVLS*(*u*, *v*) is the similarity between *u* and *v* and defined as

(1) $$\begin{array}{c} UVLS\left(u,v\right)=\frac{\left|{VLF}_{u}\cap \left.{VLF}_{v}\right|\right.}{\left|{VLF}_{u}\cup \left.{VLF}_{v}\right|\right.}, \end{array}$$

where *VLF*_*u*_ is the virtual life feature of user *u*. Higher *UVLS*(*u*, *v*) means that user *u* and user *v* have a close resemblance in regard to their virtual home style by decorating with many of the same items. The target user *u*'s *k* *VlNbrs* candidates are decided according to the descending ranking of *UVLS*(*u*, *v*).

### 3.2. User Contact Influence Analysis

In contrast to the real world, users in VWs have more interactions with strangers. Users in VWs not only engage in activities by themselves, but also interact with other users, such as visiting other users' virtual homes, sending gifts, attending a bidding on items, leaving notes, and having conversations. Users' actions and buying behaviors may be influenced by these interactions. Users may want to buy some virtual items they did not own or never considered before after visiting other's virtual homes; users may want to try something they had never noticed before after group discussions on some items. Hence, the social life circle neighbors who influence the target user's buying behaviors can be detected by analyzing the users' interactions and the influence of buying behaviors.

Users usually interact with friends in the real world; in contrast, in VWs, users may also have interactions with strangers or those who are not friends, due to fewer contact barriers. Those users who have contact with target users, no matter they are friends or not, are defined as social circle neighbors (*ScNbr*). Social circle neighbors may include the user's family members, users he visited, or the users who had visited him. More specifically, target user *u*'s social circle neighbors, *ScNbr*_*u*_, are the set of users whose frequency count of contact interactions with user *u* is above a certain threshold. The frequency count of contact interaction between users *u* and *u*_*c*_ is ∑_*sc*∈*Q*_ *cf*_*sc*_(*u*_*c*_, *u*), where *sc* is the contact activity and *Q* is a set of social interactions. Every social circle neighbor has a different influence level on the target user's buying behavior. Therefore, we have to analyze the contact strength according to the user's contact activities and contact frequency; then we measure the contact influence according to the buying influence of his neighbors.

#### 3.2.1. Contact Strength Analysis

Contact strength (CS) is the degree of intimacy between the target user and the users who influence the target user's buying behaviors after their interactions. The contact strength can be described as the activities two users have done and the frequency they interact. Analyzing users' contact strength via different interactions helps us to determine how close they are and how much the neighbor influenced the target user. Different types of interactions between the target user and his/her social circle neighbors may cause different effect of impact on the target user. The weighting factor of contact activity *sc* for target user *u*, *mp*_*sc*_^*u*^, is defined as the number of purchases influenced by *sc* divided by the total number of purchases influenced by all contact activities for user *u*. For example, after different types of interaction activities between the target user *u* and his/her social circle neighbors, the numbers of *u*'s transactions (purchases) which were influenced by visiting, chatting, and gifting are 3, 1, and 2, respectively. The influence of visiting activity is higher than that of chat activity or gift activity. Accordingly, the weighting factor for visiting, chatting, and gifting is 3/6, 1/6, and 2/6, respectively. We calculate the impact of various interactions as weights based on the number of purchases influenced by contact activities. The contact score of one interaction is defined as follows:

(2) $$\begin{array}{c} CS_{sc}\left(u,v\right)=mp_{sc}^{u}\times f_{sc}\left(u,v\right), \end{array}$$

where *f*_*sc*_(*u*, *v*) is the normalized contact count score *cf*_*sc*_(*u*, *v*) derived from the frequency count of contact activity *sc* between *u* and *v*. *mp*_*sc*_^*u*^ signifies the weighting factor of contact activity *sc*. After computing the contact strength score from each interaction, we summarized them to get a total score, and this score is the contact strength for target user *u* and the social circle neighbor user *v* and is defined as follows:

(3) $$\begin{array}{c} CS\left(u,v\right)=\underset{sc\in Q}{\sum }CS_{sc}\left(u,v\right), \end{array}$$

where *Q* is a set of social interactions and *CS*(*u*, *v*) is the summation of each social contact activity's scores between the target user *u* and interactive user *v*. Higher contact strength implies that the two users are closer to each other.

#### 3.2.2. Analyze the Contact Influence of Social Circle Neighbors

After detecting the contact strength, we then consider the contact influence on buying behaviors. Target users may buy the same virtual goods after interacting with other users. For example, when a target user visits another user's home and finds a special Christmas tree, it may occur to him/her to buy the same Christmas tree. In this example, the target user was influenced by his/her social circle neighbor. Thus, we can compute the contact influence between the target user and their social circle neighbors.

We not only consider the perspective of the influenced user, but also the influential side to improve accuracy. For instance, the influenced user (target user) *u* has 40 VLF and 5 of them were bought after contact with *v*_1_ and *v*_2_. In the influenced user *u*'s point of view, *u*'s influences from users *v*_1_ and *v*_2_ are both 5/40. But if *v*_1_'s and *v*_2_'s have 50 and 10 VLF, respectively, then *v*_2_ is more influential than  *v*_1_ because the buying influence to user *u* is 5/50 and 5/10 on the viewpoint of *v*_1_ and *v*_2_. The above situation means user *v*_2_ has more influence on user *u* than *v*_1_. In order to measure the contact influence precisely, we combine two sides of value into a linear formula to form the buying influence as follows:

(4) $$\begin{array}{c} BI\left(v,u\right)=\left(\alpha \times \frac{\left|BVI_{v\to u}\right|}{\left|BVI_{u}\right|}+\left(1-\alpha \right)\times \frac{\left|BVI_{v\to u}\right|}{\left|BVI_{v}\right|}\right), \end{array}$$

where 0 ≤ *α* ≤ 1. *BVI*_*u*_ and *BVI*_*v*_ are the sets of virtual items user *u* and user *v* bought, respectively. *BVI*_*v*→*u*_ is the set of virtual items user *u* bought after interacting with user *v*. The parameter *α* is used to adjust the relative importance between user *u*'s side and user *v*'s side. If *α* is equal to 1, the buying influence is totally derived from the view point of user *u*, and vice versa.

In addition, we add the contact strength to this method to ensure that users *u* and *v* are truly influenced and that the influence between both users is not coincidental. The contact influence score between target user *u* and his/her social circle neighbor *v* is defined as

(5) $$\begin{array}{c} CI\left(v,u\right)=BI\left(v,u\right)\times \left(1+CS\left(u,v\right)\right), \end{array}$$

where *CS*(*u*, *v*) is the contact strength computed in (3).

Recommendations can be conducted based on contact influences. The recommendation score of an item *i* for target user *u* can be derived from *u*'s social circle neighbors and their contact influence as described in

(6) $$\begin{array}{c} {CI\text{-}RS}_{u,i}=\underset{u_{c}\in ScNbr_{u},i\in VLF_{u_{c}}}{\sum }CI\left(u_{c},u\right), \end{array}$$

where *CI*-*RS*_*u*,*i*_ is the recommendation score derived based on contact influence, which considers buying influence and contact strength. ∑_*u*_*c*_∈*ScNbr*_*u*_,*i*∈*VLF*_*u*_*c*___ *CI*(*u*_*c*_, *u*) is the summed contact influences of target user *u*'s social circle neighbors who own item *i*. The more the interacted users have the item and the higher the summation is, the more likely the user *u* will buy the item *i*. *ScNbr*_*u*_ is the set of target user *u*'s social circle neighbors whose contact influence scores with user *u* are the Top-*k* highest; *VLF*_*u*_*c*__ is the set of virtual goods user *u*_*c*_ owns.

Similarly, we can derive recommendation scores based on buying influence *BI* among the target user *u*'s social contact neighbors who own item *i* as follows:

(7) $$\begin{array}{c} {CI\text{-}NCS\text{-}RS}_{u,i}=\overset{}{\underset{u_{c}\in {ScNbr}_{u},i\in {{VLF}_{u}}_{c}}{\sum }}BI\left(u_{c},u\right), \end{array}$$

where *CI*-*NCS*-*RS*_*u*,*i*_ is the item *i*'s recommendation score for target user *u* considering only buying influences. It is derived from the buying influences of social contact neighbors who own item *i* without considering the contact strengths.

### 3.3. Virtual Goods Recommendation

The target user *u*'s preference toward a virtual item *i* is combined with the similarity of his/her virtual life similar neighbors (*VlNbr*) and the contact influence derived from interactions with his/her social circle neighbors (*ScNbr*). In the goods recommendation phase, we combined the target user *u*'s virtual life similarity (UVLS) with his/her virtual life similar neighbors (*VlNbr*) and the contact influence (CI) between target user *u* and his/her social virtual circle neighbors (*ScNbr*) to obtain a virtual goods *i*'s recommendation score for user *u*. It is defined as follows:

(8) CI-UVLS-RSu,i=β×∑uc∈ScNbru,i∈VLFucCIuc,u+1−β×∑us∈VlNbru,i∈VLFusUVLS(us,u),

where *VlNbr*_*u*_ is the set of target user *u*'s virtual life similar neighbors whose virtual life similarity scores with *u* are the Top-*k* highest. ∑_*u*_*s*_∈*VlNbr*_*u*_,*i*∈*VLF*_*u*_*s*___ *UVLS*(*u*_*s*_, *u*) is the summation of the virtual life similarity of user *u*'s virtual life similar neighbors, who also owns (had purchased) virtual good *i*. The higher the score is, the more likely user *u* will buy the item. *β* is the parameter in range from 0 to 1 and is used to adjust the relative importance of *CI*(*u*_*c*_, *u*) and *UVLS*(*u*_*s*_, *u*). According to the recommendation scores, we recommend the Top-*N* virtual goods with the *N* highest scores to the target user *u*.

## 4. Experiment Evaluation

### 4.1. Experiment Setting

We collected a dataset from a popular VW website in Taiwan, http://www.roomi.com.tw/. Roomi provides a VW platform for people to interact with each other. Every user can create a new avatar to represent him/her. An avatar can build his/her own lifestyle, such as decorating a personal virtual home and planting flowers in gardens. We retrieved data from April 1, 2013, to May 20, 2013: buying log, decoration items, and interaction log, such as chats, sending gifts, mail, messages and visits, in the experiment dataset. There are 249,060 “bought” records, 462,609 decorating items, 194,003 chatting records, 151,620 gifts, 56,465 e-mails, 293,242 messages, and 11,717,979 visiting records from 4,103 users. And in this dataset, we chose users who bought over 60 items and decorated more than 55 items as our target users; 70% of the data is training sets, which implements our proposed methods and produces our recommendation list. The other 30% is randomly separated into half for parameter setting and testing data.

Precision and recall are the widely used evaluation metrics in recommender systems to evaluate the quality of recommendations. Since the *F*_1_-metric combines precision and recall, it is widely used to evaluate the quality of recommendations. This measure balances the trade-off between precision and recall by assigning equal weights to both metrics. Thus, we use the averaged *F*_1_-metric among the test users in our evaluation. Precision, recall, and *F*_1_ are defined as follows:

(9) Precision=Number  of  correctly  recommended  virtual  goodsNumber  of  recommended  virtual  goodsRecall=Number  of  correctly  recommended  virtual  goodsNumber  of  bought  virtual  goodsF1=2×recall×precisionrecall+precision.

In the experiment phase, we compared virtual goods recommendation methods to evaluate the recommendation quality. The compared methods are listed as follows.

1. User-based CF (UCF): the Jaccard similarity is used in user filtering and recommending items, as defined in (1).
2. Contact influence-based filtering enhanced with contact strength (CIF): the CIF method derives recommendation scores from target users' social circle neighbors by considering their contact influences and contact strengths in relation to the target user, as defined in (6).
3. Contact influence-based filtering without considering contact strength (CIF-NCS): the CIF-NCS method derives recommendation scores from target users' social circle neighbors by considering their buying influences in relation to the target user. The CIF-NCS method does not consider their contact strengths, as defined in (7).
4. Users virtual life feature and contact influence-based filtering (UVLF-CIF): the proposed method described in (8) combines both user similarity and contact influence-based filtering to derive recommendation scores.

### 4.2. Experiment Results

#### 4.2.1. Determine Parameters

We determined the experiment parameters in the pretest phase by using 15% of the dataset. The parameters are *α* and *VlNbr* in the UCF method, *ScNbr* in the CIF method, and the parameter *β* in UVLF-CIF method. *α* is used to weight the contact influence from the perspective of influenced user; *VlNbr* is the number of similar neighbors used in determining virtual life features; *ScNbr* is the number of social circle neighbors who interacted with the target user and influence the buying behaviors of the target user; *β* is the weight between contact influence and users virtual life similarity. After the pretest phase, *α* was set 0.9; *VlNbr* and *ScNbr* were both 60; and *β* was 0.3. The details are described in the following.

*Determining VlNbr in UCF Method*. In this experiment, we compared the recommendation quality under different numbers of similar virtual life neighbors. The UCF method recommends items to a target user based on similar users whose virtual life feature is similar to that of the target user. The number of neighbors is set from 20 to 100 in increments of 20. The highest value of *F*_1_ is located in 60 neighbors, by which we got the best recommendation quality, while the worst accuracy is located in 20 neighbors. Therefore, we used Top-60 similar users of target users to predict the recommendation score of an item via the UCF and UVLF-CIF methods.

*Determining α and ScNbr in the CIF Method*. We conducted an experiment to compare different values of parameter *α*; they range from 0 to 1 in the CIF method. The parameter *α* is used to weight the contact influence from the perspective of influenced user, as we defined in (4). When *α* is 0, the contact influence is derived entirely from the perspective of the influential user. Otherwise, the social influence is derived totally from the perspective of influenced user when *α* is 1. We conducted our experiment by systematically adjusting the value of *α* in increments of 0.1 to determine the optimal value for *α*. The optimal value of *α* which led to the highest value of *F*_1_ was chosen for the CIF method. In addition, the predicted score of a target item was derived by using the influence scores of the target user's social life circle neighbors who have bought the target item. Our experiment compared the *F*_1_ value under different settings of *α* in the CIF method. The highest *F*_1_ value occurred when *α* was 0.9. This means that the importance of the contact influence from the perspective of the influenced user is 0.9. When *α* is 0.9, the performance of the CIF method outperforms the CIF with *α* set to 0 or 1. Therefore, we set the value of *α* at 0.9 for the CIF and CIF-NCS methods to predict the recommendation score of items in the following experiments.

After determining the value of *α*, we then conduct experiment to compare the recommendation quality under different numbers of social circle neighbors. The CIF method derives recommendation scores of items based on social circle neighbors who interacted with the target user and influenced the buying behavior of the target user. The number of neighbors is set from 20 to 100 in increments of 20. The CIF method with 60 neighbors can achieve the best recommendation quality. Thus, we use Top-60 social circle neighbors of target users to predict the recommendation score of items in the CIF and CIF-NCS methods.

*Determining the Effect of Contact Strength.* The CIF method derives recommendation scores from the target user's social circle neighbors by considering their buying influences and contact strengths in relation to the target user. In order to confirm that the factor of contact strength is effective, we compared the CIF method with the CIF-NCS method without considering the contact strength by using *F*_1_-metric.

**Figure 2:**
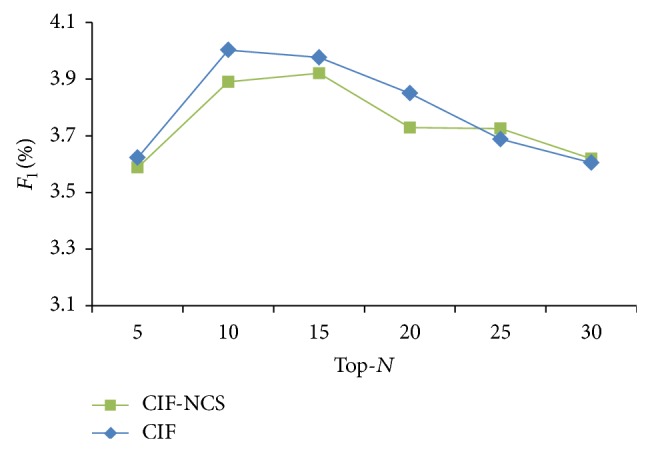
Comparing the effect of contact strength under different values of Top-*N* items.

Figure 2 shows the *F*_1_ values under different values of Top-*N* (Top-5~Top-30) items for CIF and CIF-NCS methods, respectively. The CIF method generally outperforms the CIF-NCS method. The result reveals that considering contact strength can enhance the recommendation quality of the CIF method.

**Figure 3:**
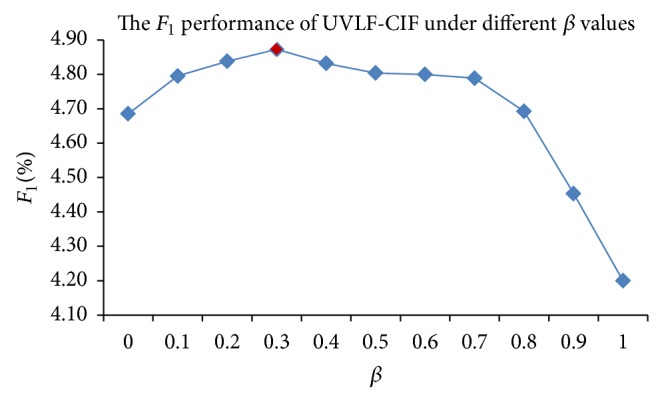
The *F*_1_-metric of *UVLF*-*CIF* method under different *β*.

*Determining β in the UVLF-CIF Method.* The UVLF-CIF method derives recommendation scores for items based on the combination of both user virtual life similarity and contact influences. To combine these models, we use a linear combination to get better performance with parameter *β*, as defined in (8). When *β* is 0, the score is derived totally from the UVLS score. When *β* is 1, the predicted score is derived entirely from the contact influence score. We set the value of *β* by systematically adjusting the value of *β* in increments of 0.1.  Figure 3 shows the *F*_1_ value under different settings of *β* in the UVLF-CIF method. When *β* is 0.3, the UVLF-CIF method gets the highest *F*_1_ value. Thus, employing both methods can improve the recommendation quality.

#### 4.2.2. Comparison of All Methods

**Figure 4:**
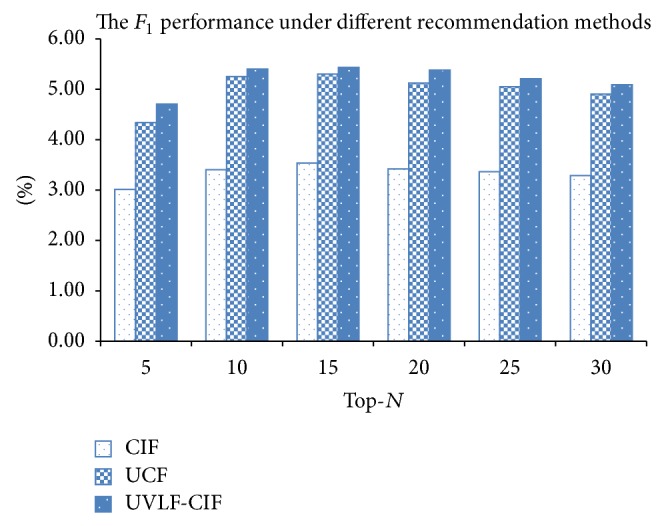
The performance under different recommendation methods.

Based on the characteristics in users' virtual lives and the contact influence from the social interaction, we proposed the UVLF-CIF method to recommend virtual goods. We compared our proposed method with traditional methods in this experiment. The UCF methods only consider user virtual life features. The UCF method uses* Jaccard similarity* to compute user similarity. The CIF method considers contact influence for buying behaviors and includes contact strength to enhance the recommendation quality.  Figure 4 depicts the recommendation performance of three methods while recommending Top-*N* items. It shows that most of the methods have best performance while recommending the Top-15 items to users; the quality of all recommendations descends when more items are recommended. Our proposed UVLF-CIF method outperforms the other methods. The result implies that considering virtual contact influences of the target user's virtual social neighbors can enhance the recommendation quality.

## 5. Conclusion and Future Works

The growing number of virtual goods in VWs entails the difficult problem of finding desired items. The users' virtual life features are distinct and visualized. Users not only interact with friends but also social life circle neighbors in VWs, and these interactions influence their buying behaviors. In this paper, we propose a novel recommendation method based on the characteristics in users' virtual life and the contact influence from the social interaction in order to recommend virtual goods in VWs. Our proposed UVLF-CIF method outperforms the other methods. The contact strengths and contact influences, which result from interactions with social circle neighbors, will influence a user's buying intention and can be used to enhance the recommendation quality for goods recommendation in VWs. The experiment results also show that considering both virtual life features and contact influence is effective in enhancing the recommendation quality and achieving better performance.

The proposed method recommends virtual goods based on users' activity history; however, it may be difficult to recommend virtual goods for new players due to the limited information of theirs. Therefore, the new players were excluded from our experiment. However, a VW platform should provide a set of virtual goods to help new users to get involved in the playground quickly and solve the cold-start problem. One of our future works is to open this limitation.

Moreover, virtual goods recommendation can be derived not only from the interaction frequency between users, but also from the interaction content. The content of players' discussion, mail, or family activities determines the accuracy of exactly what they like and care about. Retrieving more users decisive information will help to enhance the recommendation. The recommendation methods considering interaction will be investigated in our future works.
